# Synthesis of Energetic 7-Nitro-3,5-dihydro-4*H*-pyrazolo[4,3-*d*][1,2,3]triazin-4-one Based on a Novel Hofmann-Type Rearrangement

**DOI:** 10.3390/molecules26237319

**Published:** 2021-12-02

**Authors:** Fu-Qiang Bi, Yi-Fen Luo, Jun-Lin Zhang, Huan Huo, Bo-Zhou Wang

**Affiliations:** 1 Xi’an Modern Chemistry Research Institute, Xi’an 710065, China; bifuqiang@msn.com (F.-Q.B.); luoyiluoyiluoyi204@163.com (Y.-F.L.); huohuan-234@163.com (H.H.); 2 College of Chemistry & Materials Science, Northwest University, Xi’an 710127, China

**Keywords:** synthesis, NPTO, Hofmann-type rearrangement, mechanism, crystal

## Abstract

Rearrangement reactions are efficient strategies in organic synthesis and contribute enormously to the development of energetic materials. Here, we report on the preparation of a fused energetic structure of 7-nitro-3,5-dihydro-4*H*-pyrazolo[4,3-*d*][1,2,3]triazin-4-one (NPTO) based on a novel Hofmann-type rearrangement. The 1,2,3-triazine unit was introduced into the fused bicyclic skeleton from a pyrazole unit for the first time. The new compound of NPTO was fully characterized using multinuclear NMR and IR spectroscopy, elemental analysis as well as X-ray diffraction studies. The thermal behaviors and detonation properties of NPTO were investigated through a differential scanning calorimetry (DSC-TG) approach and *EXPLO5* program-based calculations, respectively. The calculation results showed similar detonation performances between NPTO and the energetic materials of DNPP and ANPP, indicating that NPTO has a good application perspective in insensitive explosives and propellants.

## 1. Introduction

**Figure 1:**
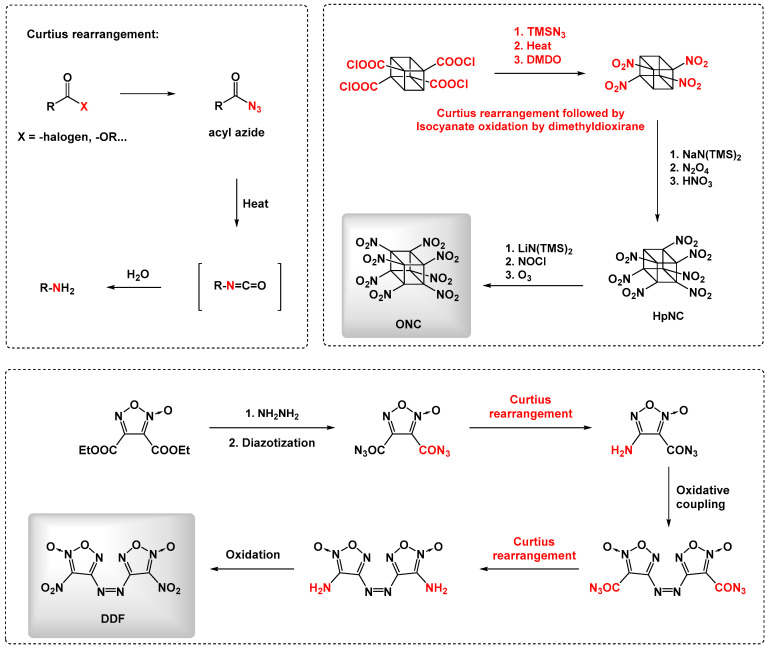
Curtius rearrangement in the synthesis of energetic materials.

As has been well-recognized, rearrangement reactions are the most efficient strategies in organic synthesis, which normally achieve the desired frameworks with remarkably high efficiency [1]. Meanwhile, rearrangement reactions are also important to the synthetic studies of energetic materials, especially for the formations of fused molecular skeletons or functional groups. For instance, an amino group (-NH_2_) is a key structural unit for the formation of energetic groups such as nitro (-NO_2_) and nitroamine (-NNO_2_) groups. Moreover, the strong hydrogen bonding effect between -NH_2_ and other O-rich groups normally plays an important role in achieving insensitivities and heat resistance properties of energetic materials [2,3]. Therefore, the introduction of -NH_2_ groups is significant in the synthesis of energetic materials. A Curtius rearrangement is one of the most effective methods to convert the carbonyl structures connected to the energetic framework into amino structures, which has been applied to the synthesis of cage-like frameworks and nitrogen-rich aromatic ring energetic materials, such as octanitrocubane (ONC) and 4,4′-dinitro-3,3′-diazenofuroxan (DDF) [4,5,6,7]. (Figure 1)

**Figure 2:**
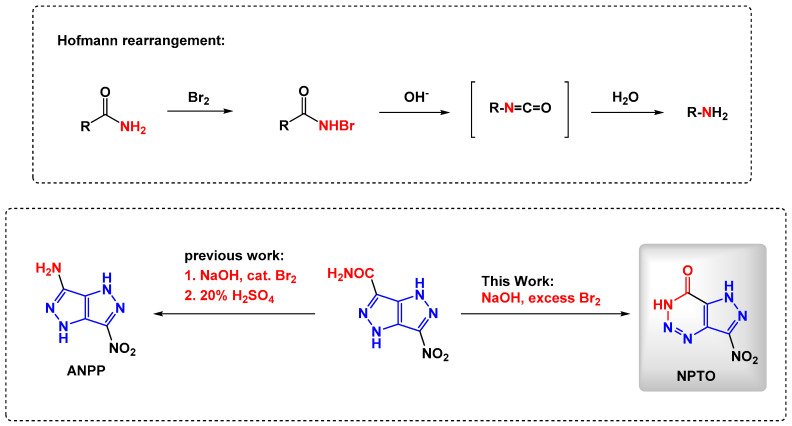
Hofmann rearrangement and the synthesis of NPTO.

Similar to a Curtius rearrangement, a Hofmann rearrangement is another widely applied reaction for the conversion of carbonyl structures to amino structures. For amide-based substrates, bromine (or chlorine) can be applied for the formation of amino structures under alkaline conditions. However, thus far, the applications of Hofmann reactions in the synthesis of energetic materials have rarely been reported, especially in the construction of new framework structures. Nitropyrazoles are significant for the design and synthesis of energetic materials [8,9]. During the past few decades, our research interest was mainly focused on the structural diversity of fused-heterocyclic frameworks in energetic materials, among which, the pyrazolo [4,3-c]pyrazole structure was a successfully applied framework in the construction of 1*H*,4*H*-3,6-dinitropyrazolo [4,3-*c*]pyrazole (DNPP), as well as its insensitive derivative [10,11,12,13], 1,4-diamino-3,6-dinitropyrazolo[4,3-c]pyrazole (LLM-119) [14], which contains both amino and nitro groups. 1*H*,4*H*-3-Amino-6-nitropyrazolo[4,3-*c*]pyrazole (ANPP) is another pyrazolo[4,3-c]pyrazole framework-based insensitive energetic material that is ideal as the key intermediate for subsequent transformations in the design and synthesis of multiple kinds of pyrazole-based energetic materials [15,16,17]. Herein, we reported a brand-new Hofmann-type rearrangement that lead to a novel fused aromatic compound of 7-nitro-3,5-dihydro-4*H*-pyrazolo[4,3-*d*][1,2,3]triazin-4-one (NPTO) during our recent studies toward ANPP through a traditional Hofmann rearrangement. (Figure 2) The new compound of NPTO was fully characterized using multinuclear NMR and IR spectroscopy, elemental analysis as well as X-ray diffraction studies. Its thermal behaviors and detonation properties were investigated through a differential scanning calorimetry (DSC-TG) approach and *EXPLO 5* program-based calculations, respectively.

## 2. Results and Discussion

### 2.1. Synthesis and Characterization towards NPTO

**Scheme 1:**
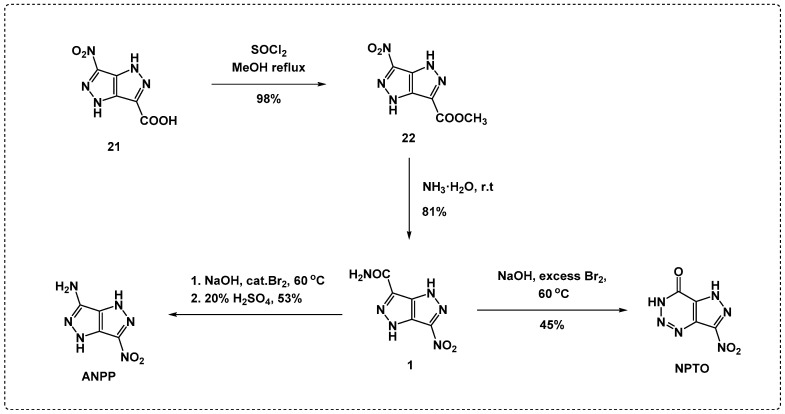
Synthetic routes towards NPTO.

Starting from 1*H*,4*H*-3-carboxy-6-nitropyrazolo[4,3-*c*]pyrazole, the synthesis of ANPP was carried out through methyl esterification, amidation and a Hofmann rearrangement with the addition of a catalytic amount of Br_2_ [18]. However, when we treated compound **1** with excess bromine in aqueous sodium hydroxide, an interesting Hofmann-type ring-expansion rearrangement was achieved, leading to the formation of NPTO with a yield of 45%. (Scheme 1, Figures S5 and S6)

**Figure 3:**
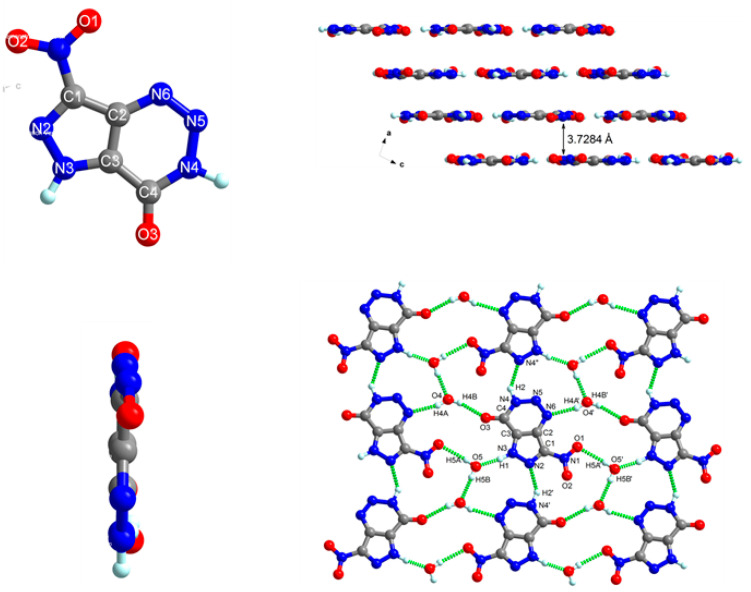
Molecular structure of NPTO∙2H_2_O and its crystal packing.

In order to explain the insensitive characteristics of NPTO from a structural point of view, NPTO was characterized through single crystal X-ray diffraction. (Figure S4) The crystal was obtained by dissolving NPTO in a mixture of ethanol/water, and detailed information on the crystallographic data is given in Table 1. The single-crystal X-ray diffraction results proved that the compound belongs to the monoclinic crystal system, space group *P*2(1)/*n*. Two molecules of water were wrapped in the crystal structure, leading to a low density of 1.693 g/cm^3^. The entire molecule presented a planar structure, with the twist angles close to 0° or 180° (O2-N1-C1-N2 (0.779°), C1-C2-N6-N5 (−178.775°)). The length of the C1-N1 bond connected to the nitro group is 1.4453 (4) Å, which is obviously shorter than that of common carbon–nitrogen bonds, indicating a strong conjugated effect [19]. The molecular stacking diagram of the crystals is a standard “layer by layer” pattern with “π-π” stacking interactions and a layer spacing of 3.7254 Å [20]. As has been proved in the insensitive structures of TATB and LLM-105, strong hydrogen bonds can significantly reduce the “hot spots” in a crystal [21,22,23,24]. There are seven types of intermolecular hydrogen bonds (N4-H2…N4’’ (3.0100 Å, 143.2°), O4-H4B...O3 (2.8837(4)Å, 162.9°), N3-H1…O5 (2.6453 Å, 170.0°), N4’-H2’…N2 (3.0100 Å, 143.2°), O5’-H5A’…O1 (2.9985 Å, 170.7°), O4-H4A’…N6 (2.8907 Å, 165.1°) and O5-H5A...O4 (2.7407(7)Å, 178.2°)), which explained the excellent insensitive performances of NPTO [25,26]. (Figure 3).

### 2.2. Studies on Thermal Behaviors of NPTO

**Figure 4:**
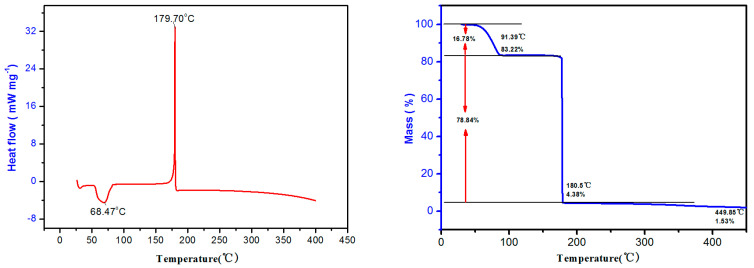
DSC-TG experiments of NPTO∙2H_2_O.

The thermal behaviors of the NPTO∙2H_2_O crystal were investigated based on the TG-DSC experiments. (Figures S1–S3) According to the experimental results, the thermal decomposition of the NPTO∙2H_2_O crystal can clearly be divided into two stages. The DSC analysis chart of NPTO is shown in Figure 1. It can be seen that NPTO has an endothermic peak in the temperature range of 52–84 °C, and the peak temperature is 68.47 °C. Combined with the structural characterization of the compound, this is the process of removing crystal water from the compound. After the loss of crystal water, the compound is relatively stable before reaching the decomposition temperature. The initial exothermic temperature of NPTO is 160.5 °C, and the exothermic peak temperature is around 179 °C. The exothermic peak of the thermal decomposition of NPTO is sharp, with a large heat release. The TG-DTG analysis chart of NPTO is shown in Figure 4. It can be seen from the Figure 4 that the thermal decomposition of NPTO is divided into at least two stages. The previous process is the process of losing crystal water, with a total weight loss of 16.78%. The sample is stable before 164 °C, and the maximum weight loss peak appears at 178 °C. When the temperature reaches 180.5 °C, the cumulative decomposition depth of the sample is 95.62%. With a further rise in the heating temperature, the substance further decomposes and finally leaves 1.53% of black “residue”.

**Figure 5:**
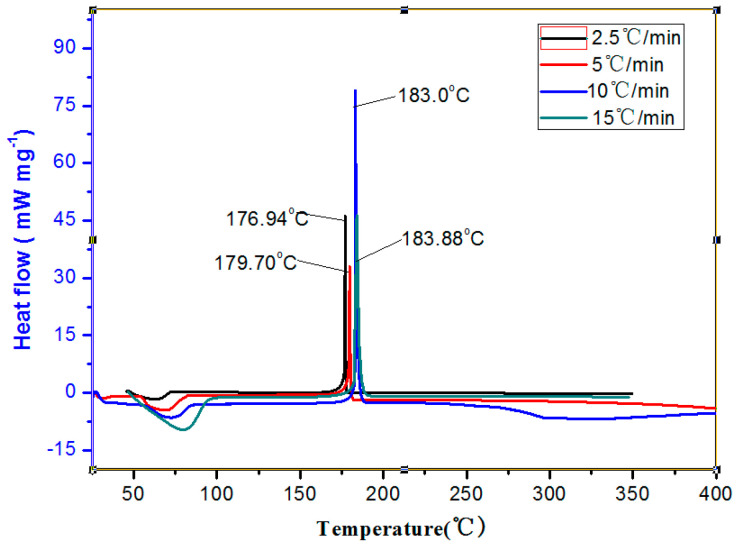
DSC experiments of NPTO∙2H_2_O at different heating rates.

The thermodynamic parameters of the thermal decomposition process of NPTO will be useful when evaluating the safety properties of the compound. (Figure 5) The Kissinger method (Equation (1)) was applied for the calculation of the thermal decomposition activation energy (*E_a_*) and the pre-exponential factor (*A*) of NPTO, where *β_i_* is the heating rate and *T_pi_* is the peak temperature at different heating rates [27]. In addition, according to *T_pi_* and *E_a_* at different heating rates, the initial thermal decomposition peak temperature *T_p_*_0_ (when the heating rate *β* approaches 0) can be obtained using Equation (2). When the heating rate *β* approaches 0, the values of *E*, *A* and *T* will be *E_a_*, *A_a_* and *T_p_*_0_. The activation enthalpy (Δ*H*^≠^) can be calculated through Equations (3) and (4), where k_B_ is Boltzmann’s constant and h is Plank’s constant. All the results are shown in Table 2. The high *E_a_* indicates a slow decomposition rate and potential long-term storage.

(1) $$ln\left(\frac{\beta_{i}}{T_{Pi}^{2}}\right)=ln\left(\frac{A_{a}R}{E_{a}}\right)-\frac{E_{a}}{RT_{Pi}}$$

(2) $$T_{Pi}=T_{P0}+a\beta_{i}+b\beta_{i}^{2}+c\beta_{i}^{3}$$

(3) $$A=\frac{k_{B}T}{h}exp\left(\frac{\Delta S^{\neq }}{R}+1\right)$$

(4) $$\Delta H^{\neq }=E-RT$$

### 2.3. ESP Analysis Studies of NPTO

**Figure 6:**
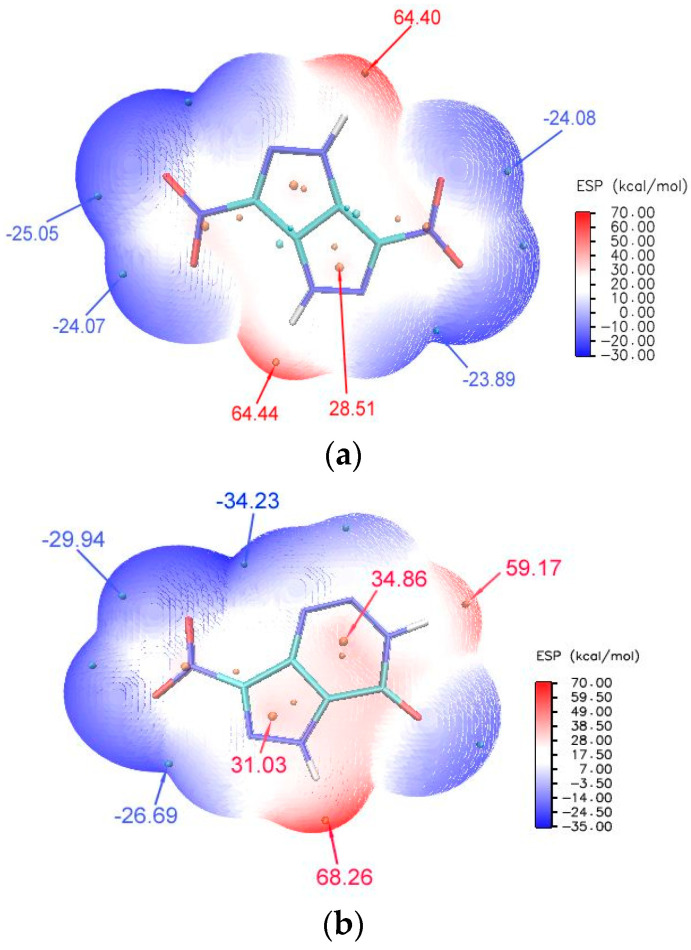
ESP-mapped surfaces of DNPP (**a**) and NPTO (**b**).

Electrostatic potential (ESP) is a real and fundamentally significant physical property of compounds as it provides information about charge density distribution and molecular reactivity [28,29,30]. Therefore, the surface electrostatic potential was taken into consideration in the analysis of the electronic properties of NPTO. The electrostatic potential of DNPP was calculated for comparison. The ESP-mapped surfaces of DNPP and NPTO are presented in Figure 6. Some pivotal maxima and minima of ESP are expressed by orange and cyan spheres, respectively. It can be seen from Figure 6 that the strongly positive ESPs distribute in the central regions of molecules and above C-NO_2_ and N-H bonds, while the negative ones concentrate on the edges of molecules, especially on the oxygen atoms of nitro groups. It was proposed by Politzer et al. [31] that the impact sensitivity of explosives has a positive correlation with the surface potential maxima (*V*_s,max_), namely, the impact sensitivity increases with a higher positive value of *V*_s,max_. The global *V*_s,max_ of NPTO (68.26 kcal/mol) is higher than that of DNPP (64.44 kcal/mol), indicating that the impact sensitivity of NPTO may be higher compared with DNPP. According to Zeman et al., the increase in negative surface potential minima (*V*_s,min_) and/or the sum of *V*_s,max_ and *V*_s,min_ (*V*_tot_) corresponds to an increase in the detonation velocity (*D*). The global *V*_tot_ of NPTO and DNPP are 34.03 and 39.39 kcal/mol, respectively. It is not hard to see that *V*_tot_ decreases as compared to DNPP. Accordingly, it can be speculated that NPTO possesses a lower detonation velocity than DNPP.

### 2.4. SEM Morphology of NPTO

**Figure 7:**
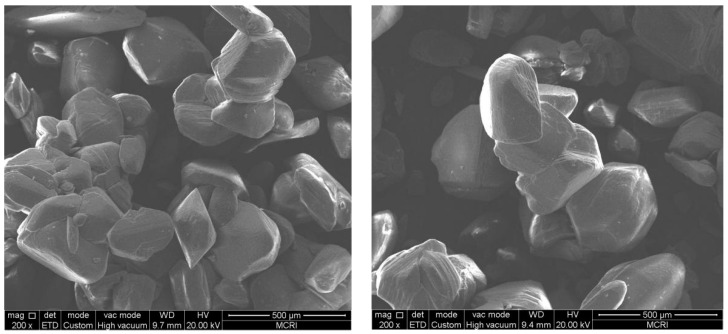
SEM Morphology graphs of NPTO.

The morphology of NPTO was examined with a HITACHI (Japan) S-3400N-II Scanning Electron Microscope (SEM) at 5 kV and 10 mA. (Figure S7) Crystal quality, including the crystal shape, surface and defects, plays an important role in the safe storage and transport of an energetic material, and ultimately affects the detonation performance of the explosive [32,33]. As shown in Figure 7, the crystal exhibits a colorless-prism-type morphology with a uniform size, regular structure and smooth surface. Considering the crystal’s approximate spherical shape, which can increase the charge density to a great degree; it is a good candidate for practical application as an energetic material.

### 2.5. Studies on Detonation Performances of NPTO

Studies on the detonation properties of the NPTO were carried out through quantum computations methods with the Gaussian 09 (Revision A. 02) suite of programs. [34] The optimized structures were characterized to be true local energy minima on the potential-energy surface without imaginary frequencies. The gas phase heats of formation were calculated with the atomization method using the Gaussian 09 program package at the b3lyp/6-311g** level of theory [34,35,36]. The gas phase heat of formation was transformed to solid phase heat of formation using Trouton’s rule [37,38]. Based on the density and heat of formation calculated, the detonation properties for NPTO were calculated by EXPLO5 6.04 [39]. All the data are summarized in Table 3.

NPTO showed great insensitivities toward the impact and friction stimulations. From a structural point of view, the aromatic system size can significantly affect the characteristics of highly energetic compounds, especially their sensitivity toward detonation. This influence usually results in a decrease in the sensitivity of these compounds toward detonation. Typical examples include the insensitive explosives of 2,2′,4,4′,6,6′-hexanitrostilbene (HNS) and 2,2,4,4,6,6-hexanitroazobenzene (HNAB), whose conjugation between aromatic rings have been well known to increase stability in explosives. Obviously, the conjugation between aromatic rings of NPTO also played a similar role, leading to its excellent insensitivities toward external stimulations.

## 3. Conclusions

In this study, we achieved the synthesis of NPTO based on a unique Hofmann-type reaction. This synthetic strategy is the first time that a Hoffmann-type reaction has been used for the construction of a fused skeleton in the research field of energetic materials. Through NMR, elemental analysis, X-single crystal diffraction and other means, the structure of NPTO has been fully characterized. DSC-TG studies showed NPTO has good thermal stability. Further theoretical studies have further shown that the energy density level of NPTO is close to that of DNPP, while the sensitivity level is better than that of DNPP, making it an ideal candidate for the develop of insensitive explosives.

## Figures and Tables

**Table 1:** Crystallographic details of NPTO∙2H_2_O.

Compound	NPTO∙2H_2_O
Formula	C_4_H_6_N_6_O_5_
Formula weight	218.15
T (K)	296(2)
λ (Å)	0.71073
Crystal system	Monoclinic
Space group	*P*2(1)/*n*
a (Å)	6.608(3)
b (Å)	9.330(4)
c (Å)	13.998(6)
Volume (Å^3^)	855.7(7)
Z	4
Dc (g/cm^3^)	1.693
F (000)	448
θ range/(°)	2.63 to 25.09
Reflections collected/unique	4177/1519 [R_(int)_ = 0.0623]
Refinement method	Full-matrix least-squares on F^2^
GOF on F^2^	1.002
Final R indexes (I > 2σ(I))	R_1_ = 0.0560, *w*R_2_ = 0.1254
Final R indexes (all data)	R_1_ = 0.1058, *w*R_2_ = 0.1498
Largest diff peak and hole (e Å^−3^)	0.322 and −0.222
GOF on F_2_	0.949
CCDC number	2082280

**Table 2:** Thermodynamic parameters of the thermal decomposition process of NPTO.

Heating Rates *β*/(°C/min)	*T_pi_*/(°C)	*E_a_*/(kJ·mol^−1^)	lg(*A_a_/*s^−1^)	*T_p_*_0_/ (°C)	Δ*H*^≠^/ (KJ·mol^−1^)
2.5	176.94	429.14	27.33	173.33	427.70
5	179.7				
10	183.0				
15	183.88				

**Table 3:** Calculated physico-chemical properties and detonation parameters of NPTO.

Compound	*ρ*^a^ (g⋅cm^−3^)	Δ*H_f_* ^b^ (kj⋅mol^−1^)	*D*^c^ (m⋅s^−1^)	*P*^d^ (Gpa)	*H_50_*^e^ (cm)	*H_50_*^f^ (J)
ANPP	1.678	283.28	7317	19.79	44.63	10.94
DNPP	1.758	290.44	8068	26.96	33.98	8.32
NPTO	1.752	217.13	7556	22.32	40.44	9.91

^a^ Crystal density. ^b^ Molar enthalpy of formation. ^c^ Detonation velocity. ^d^ Detonation pressure. ^e^ Impact sensitivity. ^f^ Friction sensitivity.

## Data Availability

Not applicable.

## Supplementary Materials

The following are available online, Figure S1: DSC curve of NPTO under nitrogen with a heating rate of 5 °C·min^−1^; Figure S2: TGA curve of NPTO under nitrogen with a heating rate of 5 °C·min^−1^; Figure S3: DSC curves of NPTO under nitrogen with different heating rates; Figure S4: Molecular structure of NPTO∙2H_2_O and its crystal packing; Figure S5: ^15^N spectra of NPTO in *d*_6_ DMSO; Figure S6: 13C spectra of NPTO in d6 DMSO; Figure S7: SEM Morphology graphs of NPTO.
