# Supplementary material for: Synthesis of Energetic 7-Nitro-3,5-dihydro-4H-pyrazolo[4,3-d][1,2,3]triazin-4-one Based on a Novel Hofmann-Type Rearrangement
Source: Molecules. 2021 Dec 2;26(23):7319. doi: 10.3390/molecules26237319 (PMC8659268; doi:10.3390/molecules26237319)
Supplement: Supplementary file 1 [file molecules-26-07319-s001.zip › molecules-1449166-supplementary.pdf]

Supporting Information

Synthesis of Energetic 7-Nitro-3,5-dihydro-4H-pyrazolo[4,3-d][1,2,3]triazin-4-one based on a Novel Hofmann-type Rearrangement

Fuqiang Bi [a], Yifen Luo [a], Junlin Zhang [a,b]*, Huan Huo [a], Bozhou Wang [a,*]

a. Xi'an Modern Chemistry Research Institute, Xi'an, 710065, China

b. College of Chemistry & Mateirals Secience, Northwest University, Xi'an 710127, China

E-mails: junlin-111@163.com (Dr. Junlin Zhang), wbz600@163.com (Dr. Bozhou Wang)

Table of Contents

1. Experimental Section 3
2. Thermogravimetric analysis (TG) and differential thermal gravity (DTG) curves of NPTO. 4
3. Single-crystal X-ray diffraction analysis of NPTO 5
4. ^{15}N NMR and ^{13}C NMR spectra of NPTO 5
5. SEM Morphology of NPTO...7
6. Coordinates of the structures optimized using Gaussian software. 7

1. Experimental Section

Safety precautions! Although none of the compounds described herein has exploded or detonated in the course of this research, these materials should be handled with care using the best safety practices.

General

1H,4H-3-carboxy-6-nitropyrazolo [4,3-c]pyrazole was supplied by Xi'an Modern Chemistry Research Institute. All reagents and solvents were purchased from Aladdin Bio-Chem Technology CO., Ltd (Shanghai, China) and were used without further purification unless otherwise indicated. ^{13}C spectra were measured with AV 500 NMR spectrometer (Bruker, Switzerland). ^{15}N spectra were measured with AV 800 NMR spectrometer (Bruker, Switzerland). Infrared spectra were was measured by an EQUINOX 55 Fourier transform Infrared spectrometer (Bruker, Germany). Elemental analyses were performed with the vario EL cube elemental analyzer (Elmentar, Germany).The thermal analysis experiment and the glass transition temperature (Tg) were performed with a model TG-DSC STA 499 F3 instrument (NETZSCH, Germany). Single crystal X-ray experiment was carried out on a Bruker Apex II CCD diffractometer equipped with graphite monochromatized Mo Kα radiation (λ = 0.71073 Å) using ω and φ scan mode. Structures were solved by the direct method using SHELXTL and refined by means of full-matrix least-squares procedures on *F2* with the programs SHELXL-97. All nonhydrogen atoms were refined with anisotropic displacement parameters. The morphology was examined with a HITACHI (Japan) S-3400N-II Scanning Electron Microscope (SEM) at 5 kV and 10 mA. The sensitivity towards impact (IS) and friction (FS) were determined according to BAM standards.

Synthetic procedures

1H,4H-3-methoxycarbonyl-6-nitropyrazolo[4,3-c]pyrazole: Thionyl chloride (3mL) was added with vigorous stirring to a suspension of 1H,4H-3-carboxy-6-nitropyrazolo[4,3-c]pyrazole (1.9 g, 9.64 mmol) in methanol (40mL) at 20°C. The mixture was refluxed for 3h, cooled, and concentrated to 10mL, and the precipitate was filtered and dried on air to yield 2.0 g in yield of 98.3% as an off-white solid. ^{13}C NMR (DMSO-d_6, 125 MHz), δ: 161.049, 139.592, 137.711, 130.749, 124.897, 52.582. IR (KBr) cm^{-1}: 3302, 3119, 2964, 2859, 1698, 1550, 1524, 1500, 1454, 1421, 1372, 1232, 1129, 1039, 936, 820, 779, 734. Elemental analysis (%) calculated for $C_6H_5N_5O_4$ (M=211.03): C, 34.13; H, 2.39; N, 33.17. Found: C, 33.91; H, 2.625; N 33.42.

1H,4H-3-carbamoyl-6-nitropyrazolo[4,3-c]pyrazole: At room temperature, 1H,4H-3-methoxy carbonyl-6-nitropyrazolo[4,3-c]pyrazole (0.4 g, 1.89 mmol) was dissolved in aqueous ammonia (30 mL). The mixture was stirred for 6h and left overnight. It was concentrated to half its volume and acidified by 20% H_2SO_4 to pH 1. The precipitate was filtered, washed with water, and dried on air to yield 0.3 g in yield of 80.6% as a white solid. ^{13}C NMR (DMSO-d_6, 125 MHz), δ:

162.414, 139.377, 137.516, 130.927, 129.324. IR (KBr) cm^{-1}: 3412, 3287, 1674, 1645, 1602, 1550, 1515, 1417, 1374, 1241, 1183, 1133, 1074, 1027, 807, 730, 696. Elemental analysis (%) calculated for $C_5H_4N_6O_3$ (M=196.03): C, 30.62; H, 2.06; N, 42.85. Found: C, 30.17; H, 2.346; N, 42.22.

7-nitro-3,5-dihydro-4H-pyrazolo[4,3-d][1,2,3]triazin-4-one: Bromine was added to a solution of NaOH (6 g, 150 mmol) in water at 0~5℃ and stirred for 10min. 1H,4H-3-Carbamoyl-6-nitropyrazolo[4,3-c]pyrazole was added at 0~5℃ to the obtained solution of hypobromite. The mixture was stirred 50min, and then heated to 60℃ and kept at this temperature for 3h. Then it was cooled, acidified by 20% H_2SO_4 to pH 1. The precipitate was filtered, and dried on air to yield 0.4 g in yield of 44.9% as a colorless solid. ^{13}C NMR (DMSO-d_6, 125 MHz), δ: 149.151, 147.619, 129.547, 128.514. ^{15}N NMR (DMSO-d_6, 81 MHz), δ: 411.217, 355.943, 346.953, 315.583, 237.319, 198.468. IR (KBr) cm^{-1}: 3572, 3472, 3256, 1708, 1644, 1547, 1511, 1469, 1408, 1382, 1367, 1326, 1237, 1151, 1027, 949, 836, 779, 649. Elemental analysis (%) calculated for $C_4H_2N_6O_3{\cdot}2H_2O$ (218.04): C, 22.03; H, 2.77; N, 38.53. Found: C, 21.94; H, 2.891; N, 38.93. HRMS m/z (M - H)$^-$： calcd 181.01156, found 181.01126.

2. Thermogravimetric analysis (TG) and differential thermal gravity (DTG) curves of NPTO.

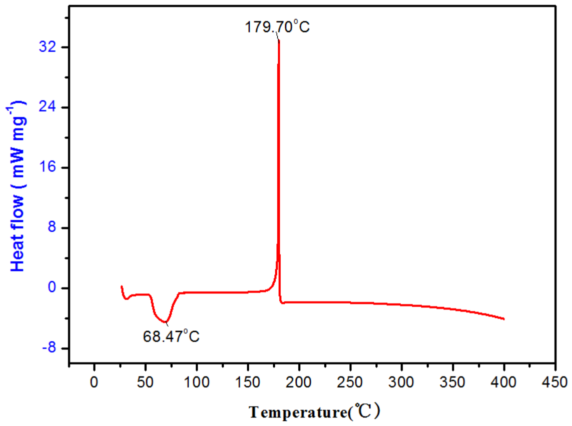

Figure S1 DSC curve of **NPTO** under nitrogen with a heating rate of 5 °C·min^{-1}

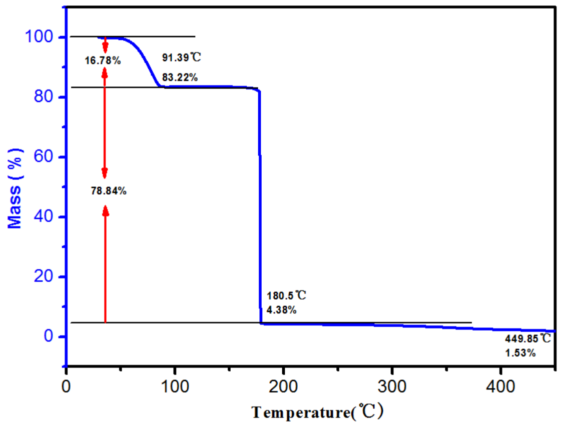

Figure S2 TGA curve of **NPTO** under nitrogen with a heating rate of 5 °C·min^{-1}

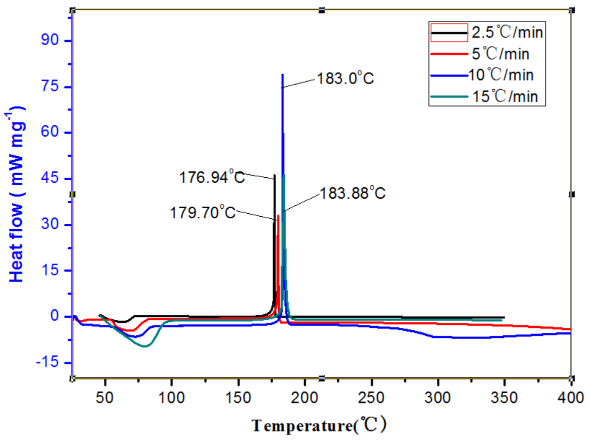

Figure S3 DSC curves of **NPTO** under nitrogen with different heating rates

3. Single-crystal X-ray diffraction analysis of NPTO·2H_2O

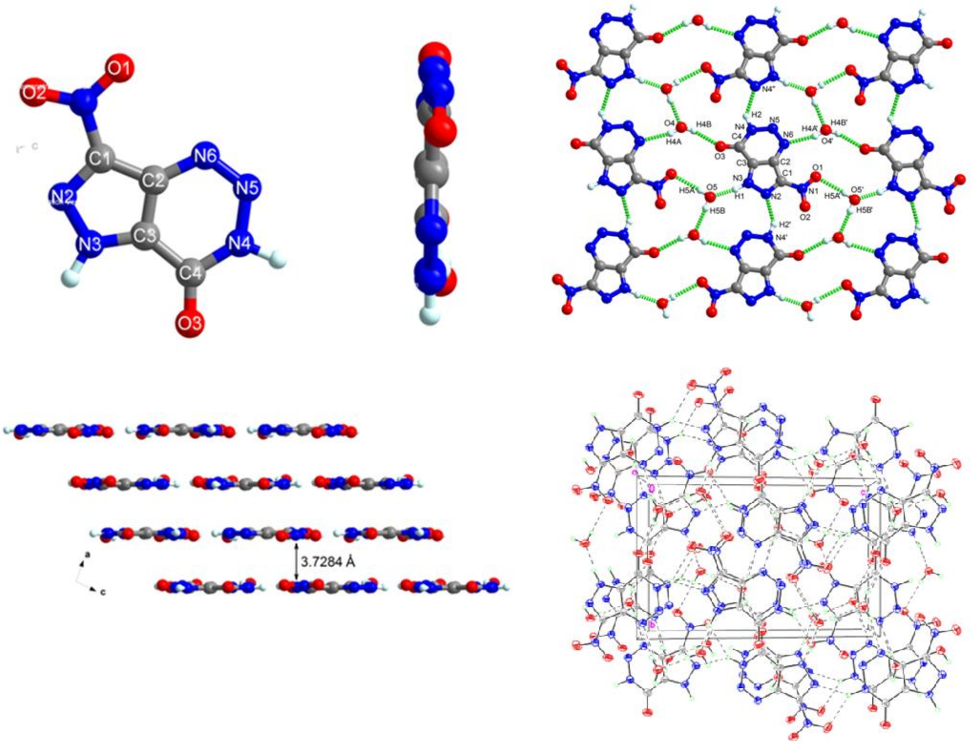

Figure S4. Molecular structure of NPTO·2H_2O and its crystal packing

4. ^{15}N NMR and ^{13}C NMR spectra of NPTO

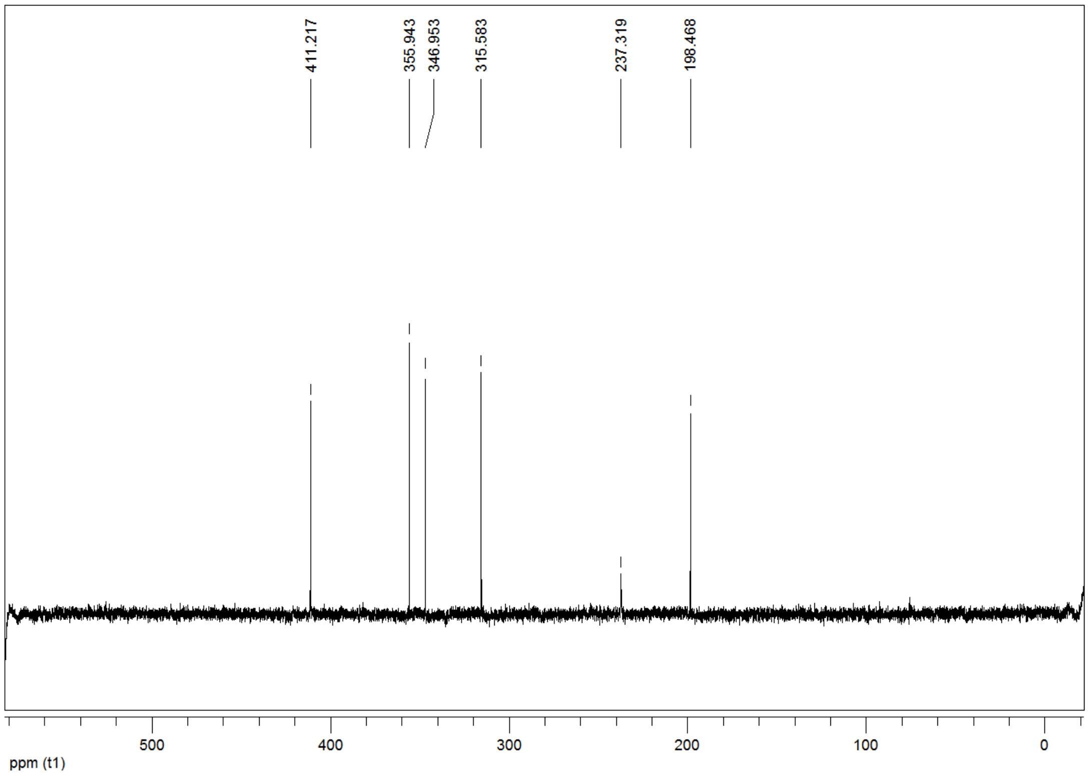

Figure S5 ^{15}N spectra of NPTO in *d*6-DMSO

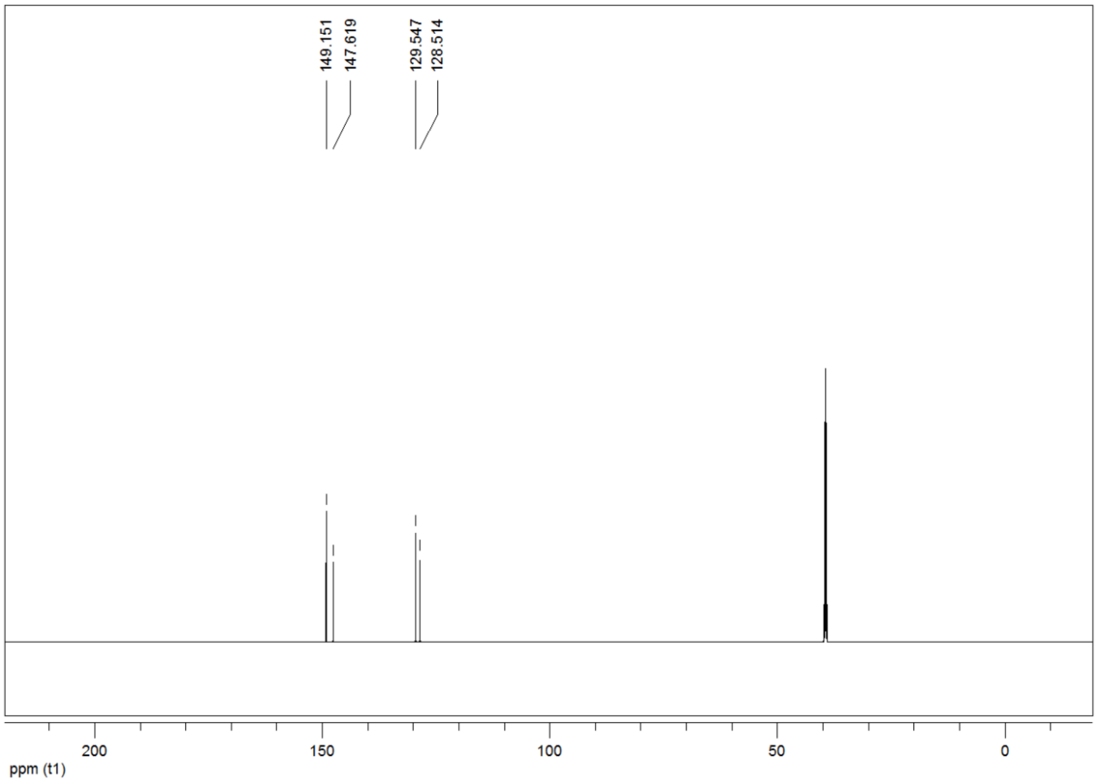

Figure S6 ^{13}C spectra of NPTO in *d*6-DMSO

5. SEM Morphology of NPTO

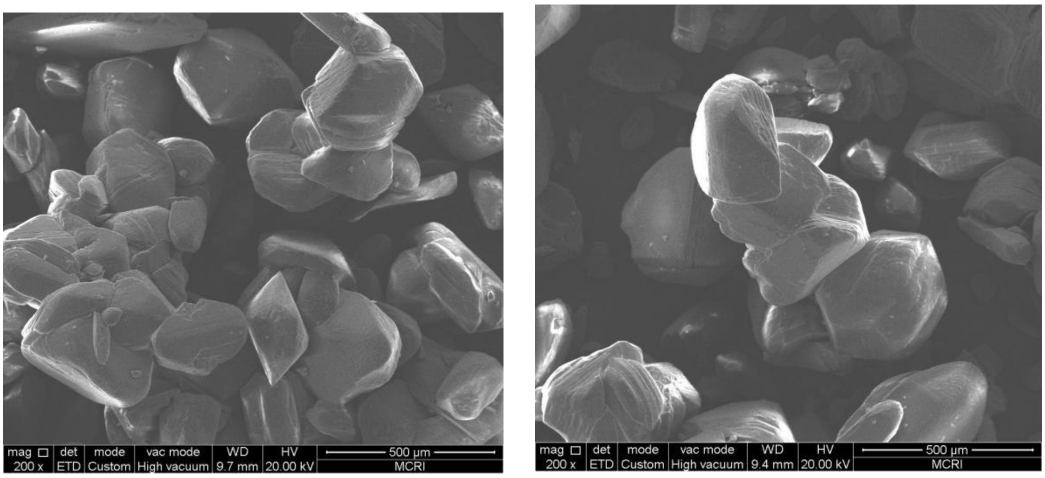

Figure s7. SEM Morphology graphs of NPTO

6. Coordinates of the structures optimized using Gaussian software

1.

C	-1.67839900	-0.28411700	0.00014600
C	0.55808800	-0.46653500	0.00004400
C	-0.47539900	0.46691500	0.00006500
C	1.73694400	0.31143100	-0.00000500
H	-0.32794600	2.60632400	0.00007100
N	-0.03070700	-1.69086100	0.00010100
N	-1.37751000	-1.58664100	-0.00002100
N	0.11685300	1.69754600	0.00004000
N	1.45148200	1.61735100	-0.00001100
H	0.40475800	-2.60279800	0.00013700
C	-3.05963400	0.25561300	0.00029100
O	-3.23338200	1.47773300	-0.00009600
N	-4.05151900	-0.66095400	-0.00019200
H	-5.01394800	-0.35342100	-0.00052100
H	-3.85114800	-1.65146200	-0.00027200
N	3.08424200	-0.16373200	-0.00002700
O	4.01185800	0.64432600	-0.00009100
O	3.21512400	-1.39548900	-0.00005200

2.

C	-1.74265400	0.37696700	-0.00004600
C	0.51721100	0.51387600	0.00003900
C	-0.54512000	-0.38528200	0.00007000
C	1.66787800	-0.30791300	-0.00007000
H	-0.50184400	-2.51283500	0.00032100
N	-0.04378100	1.75048500	0.00012800
N	-1.41043700	1.67015000	-0.00010700
N	0.00170100	-1.63504300	0.00024400
N	1.33592200	-1.61005500	-0.00037600
H	0.40856200	2.65251700	0.00012800
C	-3.12788300	-0.23136000	-0.00005000
O	-3.09156100	-1.50477400	0.00020000
N	-4.20060700	0.53033500	-0.00018000
H	-3.89394100	1.50776100	-0.00014000
N	3.02834000	0.11229200	-0.00002600
O	3.92787600	-0.73097500	-0.00006700
O	3.21276700	1.33921000	0.00014700

3.

C	0.18913500	-0.19923200	0.07224200
C	-2.03664900	-0.45124400	0.01588600
C	-1.03491800	0.51665400	0.04643100
C	-3.24045600	0.28707800	-0.01194500
H	-1.25604200	2.65111200	0.04875000
N	-1.40927600	-1.65688600	0.02343100
N	-0.07047300	-1.51158100	0.05681700
N	-1.66772300	1.72690500	0.03591300
N	-2.99835200	1.60025500	0.00068200
H	-1.81655000	-2.58195600	0.00423800
C	1.55446500	0.37605800	0.09074600
O	1.72553300	1.58729300	0.02975600
N	2.53994700	-0.56336800	0.23727900
H	2.34169700	-1.53426600	0.01325500
N	-4.57162000	-0.23350000	-0.05060300
O	-5.52344900	0.54442400	-0.07041800
O	-4.65848200	-1.46837700	-0.06154600
Br	4.37243100	-0.07286500	-0.07581100

4.

C	0.17429700	0.64688700	0.22592600
C	-2.05141300	0.48316000	-0.06694400
C	-0.92082400	-0.26714900	0.24515500
C	-3.11485200	-0.44458200	-0.03289100
H	-0.81374200	-2.37111200	0.67728300
N	-1.62358500	1.75831600	-0.26798200
N	-0.28836500	1.86445800	-0.10163100
N	-1.35351200	-1.54753000	0.44331600
N	-2.67513100	-1.66779500	0.27616300
H	-2.16682600	2.57391600	-0.51715500
C	1.63288300	0.47201300	0.87631700
O	1.95042800	0.59856000	2.04609000
N	1.90707600	0.20173300	-0.34272100
N	-4.49784100	-0.18285400	-0.28193600
O	-5.30966800	-1.10383200	-0.20896100
O	-4.77501800	0.99136600	-0.56044200
Br	4.38438800	-0.35479500	-0.45529900

5.

C	-0.27846000	1.09662200	0.00043900
C	-2.42660100	0.46339000	-0.00025400
C	-1.12759300	-0.04168200	0.00111700
C	-3.25091100	-0.68433900	0.00035900
H	-0.51337900	-2.10612300	0.00376600
N	-2.32227400	1.81311700	-0.00159800
N	-1.01986900	2.20663500	-0.00119200
N	-1.24421300	-1.40616600	0.00241600
N	-2.51896400	-1.80334100	0.00197300
H	-3.05565100	2.50713900	-0.00283700
N	-4.67758500	-0.71619700	-0.00067800
O	-5.26048300	-1.79991900	-0.00003200
O	-5.23403000	0.39098700	-0.00222300
N	1.10049100	1.10669500	0.00136100
C	1.96113800	1.96394500	0.00161400
O	2.88902500	2.68417100	0.00190900
Br	4.85498300	-1.02273500	-0.00096500

6.

C	-1.36848500	-0.40762300	0.03560400
C	0.84423500	-0.55653000	0.02784600

C	-0.19548100	0.36655300	0.00995100
C	2.00588000	0.25112000	-0.01827700
H	-0.24645800	2.44277100	-0.07895700
N	0.26601900	-1.78816700	0.06655000
N	-1.09353500	-1.70426400	0.07117700
N	0.35582000	1.60706300	-0.04692100
N	1.68831700	1.55088300	-0.06397200
H	0.71104300	-2.69323300	0.08819400
N	3.35730100	-0.22820700	-0.01756000
O	4.28393300	0.57035900	-0.05734700
O	3.46159400	-1.46419200	0.02511400
N	-2.67594800	0.13873600	0.01918200
C	-3.74610400	-0.49694500	-0.06348500
O	-4.81929800	-0.93708600	-0.12485800
O	-2.17518200	2.37768000	0.02243700
H	-2.59440200	1.17196100	0.02151300
H	-2.40462900	2.73265500	0.89745100

7.

C	-1.23387700	-0.03936500	0.02587500
C	0.98632700	-0.37492100	0.01139100
C	0.02392900	0.63314100	0.01054900
C	2.21965700	0.31492500	-0.00422100
H	0.35118300	2.76622800	-0.01348100
N	0.32145600	-1.55006600	0.02584100
N	-1.03330100	-1.35432100	0.03569600
N	0.71163300	1.82080300	-0.00511200
N	2.03307500	1.64012100	-0.01415300
H	0.68874200	-2.48948900	0.02516200
N	3.52456800	-0.25897200	-0.00959100
O	4.51142600	0.47728600	-0.02231600
O	3.56282500	-1.49800900	-0.00077500
N	-2.48720400	0.56728500	0.03340500
C	-3.70952100	-0.05986700	-0.01531600
O	-3.91489000	-1.25643600	-0.07058300
O	-4.68551700	0.88261700	0.00772900
H	-2.50790600	1.57738600	0.07222000
H	-5.53346200	0.40477400	-0.02860200

8.

C	-1.04371000	-0.42256500	-0.41479600
C	1.15554800	-0.55537500	-0.14635500
C	0.10470200	0.35281000	-0.19105900
C	2.29176800	0.26572700	0.04995500
H	-0.04013500	2.40742700	-0.08657100
N	0.60058900	-1.78583800	-0.33712500
N	-0.74370200	-1.71184100	-0.50547800
N	0.62101000	1.59835900	-0.03598200
N	1.94622500	1.55977500	0.10918900
H	1.05801800	-2.68510200	-0.35570200
N	3.64380000	-0.19378300	0.16693200
O	4.54724600	0.61384800	0.34172700
O	3.77727000	-1.42482900	0.07419700
N	-2.38178700	0.10315700	-0.56761900
C	-3.36420100	-0.37080900	0.40015700
O	-3.11584800	-0.59776300	1.54818900
O	-4.55747400	-0.42052500	-0.20827600
H	-2.74546100	-0.03142600	-1.51637900
H	-5.21782500	-0.67472100	0.46322300
O	-1.81215000	2.38540300	-0.32337800
H	-2.22814600	1.15902300	-0.43693800
H	-2.20639700	2.76818100	0.47586000

9.

C	-0.10648900	2.08640700	0.00226000
C	1.01283200	0.12453000	0.00079900
C	1.19684500	1.50841200	-0.00029000
C	2.32598400	-0.40129600	-0.00065300
H	3.05990200	2.59408800	0.00023800
N	-0.31211000	-0.10624800	0.01345600
N	-0.99469600	1.08921800	0.00276200
N	2.55250100	1.71855600	-0.00300100
N	3.24070600	0.57835200	-0.00380300
H	-0.87327900	-0.95606300	0.00282600
N	2.70062900	-1.77492500	-0.00072100
O	3.89470400	-2.07960000	-0.00226700
O	1.76249000	-2.58612300	0.00163100
N	-0.47034900	3.41573500	0.08401600
C	-3.66472400	-0.86165300	-0.00295300

O	-2.69852600	-1.61725600	-0.00720000
O	-3.63847600	0.45642500	0.00500000
H	-1.43943000	3.58596900	-0.16374500
H	-2.67756400	0.77422200	0.00606900
O	-4.93501700	-1.29410400	-0.00620400
H	0.14970200	4.05126800	-0.40531000
H	-4.90417600	-2.26744700	-0.01170000

10.

C	-1.78763800	0.16220000	-0.04052100
C	0.46125800	-0.34977900	-0.00465100
C	-0.43365300	0.71608100	-0.01366600
C	1.73852700	0.25888900	0.01305000
H	0.01912700	2.81418100	0.00275600
N	-0.27008300	-1.48414900	0.04839400
N	-1.64673500	-1.19408500	-0.04665300
N	0.32736900	1.85018900	0.00309600
N	1.64041300	1.60882000	0.01346900
H	0.04147200	-2.39734900	-0.24736900
N	2.98750500	-0.39430500	0.00974500
O	4.03675000	0.26818200	0.03252200
O	2.95188300	-1.64250000	-0.01182500
N	-2.97016200	0.77883000	-0.05545400
O	-5.42471000	-0.44245900	-0.03015500
H	-4.51755500	-0.00681100	-0.07363300
H	-5.56714800	-0.54567900	0.92270500
H	-2.83639500	1.78841300	-0.06223800

11.

C	-0.75845700	1.48519500	0.26496500
C	-2.70217500	0.45462500	-0.23039500
C	-1.60351500	0.38845500	0.62183600
C	-3.51908100	-0.63005600	0.15814100
H	-1.22736800	-1.04943600	2.17708500
N	-2.50671600	1.52640400	-1.03801500
N	-1.32573000	2.14256000	-0.75888900
N	-1.81480900	-0.69125400	1.43512200
N	-2.96514300	-1.31447700	1.16683700
H	-3.11100800	1.89468400	-1.75799300
N	-4.77456100	-1.00523100	-0.40448400

O	-5.37324900	-1.98351000	0.04327400
O	-5.17853500	-0.28966300	-1.33264900
N	0.40383600	1.90288600	0.88514300
H	0.68870700	2.78131600	0.44581100
Br	2.07369700	0.67110000	0.33387100
Br	4.51045100	-1.05843400	-0.46064800

12.

C	1.49453300	0.66674200	-0.36201000
C	-0.77459500	0.57040200	-0.08367900
C	0.35762800	-0.22241500	-0.24977400
C	-1.83563100	-0.36130800	0.03125300
H	0.51263100	-2.33852800	-0.33174700
N	-0.37017900	1.85676500	-0.15985900
N	1.03200100	1.94175200	-0.27312700
N	-0.06732400	-1.51544700	-0.23366500
N	-1.38333000	-1.63630900	-0.05954300
H	-0.86912500	2.66309900	0.18390600
N	-3.19798400	-0.07032200	0.23349700
O	-4.02573100	-0.99231900	0.31778500
O	-3.49429800	1.14121800	0.31364200
N	2.78485700	0.33892700	-0.53888600
H	4.06407100	0.31090800	0.70200200
O	2.81385500	-1.13432700	-0.63665400
H	3.51008100	-1.34108000	0.01834900
O	4.73185400	-0.19850300	1.24580900
H	5.53899400	-0.15102900	0.70917500

13.

C	-1.62675500	0.07805600	-0.36157800
C	0.64596400	-0.38359900	-0.15860900
C	-0.26210100	0.65742300	-0.26583200
C	1.90172000	0.25443200	-0.01432300
H	0.12352500	2.75378700	-0.21386000
N	-0.04316300	-1.54280000	-0.33610000
N	-1.44326900	-1.26457700	-0.45136300
N	0.46625100	1.80451500	-0.18637100
N	1.78298600	1.60405000	-0.03150800
H	0.21283700	-2.40834700	0.11667900
N	3.15640300	-0.37672000	0.16980500

O	4.18830900	0.30342200	0.24784900
O	3.13282700	-1.62279800	0.23801200
N	-2.79607400	0.72167300	-0.66890800
H	-4.11296800	-0.39838500	-0.27408600
O	-2.48902700	0.77797700	0.85838200
H	-3.09450100	0.06959600	1.18501700
O	-4.78335700	-0.74818000	0.40307700
H	-5.33380800	0.03912800	0.54086100
14.			
C	-1.71965200	0.23944700	0.12817200
C	0.56795800	-0.31831500	0.11085100
C	-0.32222500	0.73973500	0.07360100
C	1.82994300	0.29382400	-0.04481300
H	0.09007100	2.82487100	-0.10400500
N	-0.06306400	-1.55547800	0.19069400
N	-1.55079500	-1.23992300	0.37676100
N	0.41579800	1.86755900	-0.07302000
N	1.73921500	1.63936300	-0.13678000
H	0.24062600	-2.12906300	0.97566500
N	3.09438400	-0.35448400	-0.06073800
O	4.11556900	0.30425000	-0.28934500
O	3.09026100	-1.57911600	0.14940400
N	-2.09130100	-0.66942700	-0.87243500
H	-3.78668500	-0.63767700	-0.67354100
O	-2.61671600	1.03180600	0.83445500
H	-3.51398900	0.70808000	0.56734300
O	-4.70890600	-0.32699700	-0.35379700
H	-5.01748600	0.25283200	-1.06943900
15.			
C	1.76187100	0.31016300	-0.14827900
C	-0.55878800	-0.31097700	-0.22228700
C	0.35543900	0.73901900	-0.21083200
C	-1.80192300	0.31673200	0.02333600
H	-0.03023200	2.81878500	0.02305600
N	-0.01680300	-1.52829200	-0.55426500
N	1.47941000	-1.39832500	-0.62728200
N	-0.37459200	1.87154700	-0.02079600
N	-1.69673600	1.66447600	0.12446900

H	-0.33800100	-2.32960000	-0.01311000
N	-3.05716400	-0.33201900	0.16143300
O	-4.08283100	0.34143200	0.32100000
O	-3.04177100	-1.57686200	0.11390200
N	2.00281400	-0.78862300	0.64991900
H	3.66212700	-0.78003300	0.63966700
O	2.70521600	1.18155600	-0.60284600
H	3.57320300	0.79409600	-0.30084100
O	4.61227400	-0.35857000	0.59215500
H	4.69170100	0.04534000	1.47155700

16.

C	-2.34111000	-0.36155100	0.00011300
C	0.05143900	0.27784100	-0.00009600
C	-0.92760400	-0.72339400	-0.00005300
C	1.29226900	-0.43187500	-0.00004600
H	-0.69535600	-2.81344300	-0.00018200
N	-0.27672000	1.57239400	-0.00010700
N	-1.56383100	2.06696100	0.00007600
N	-0.27114000	-1.89494300	-0.00007200
N	1.07714200	-1.79920400	-0.00003200
H	0.47631200	2.25200400	-0.00007000
N	2.50649500	0.18063700	0.00002200
O	3.59081100	-0.49054200	0.00006900
O	2.49616100	1.47790900	0.00008600
N	-2.48210700	1.00468400	-0.00013600
H	-3.43598900	1.34634700	0.00001300
O	-3.30244800	-1.14545900	0.00015500

17.

C	-2.52087700	-0.31708600	-0.00025200
C	-0.04677800	-0.01720600	-0.00237500
C	-1.18387300	-0.86716700	0.00182800
C	1.07599100	-0.96004700	0.00039400
H	-1.33600200	-2.95437600	0.01084900
N	-0.12995300	1.31387400	-0.00737100
N	-1.32750100	1.92206700	-0.00879600
N	-0.75134100	-2.12981800	0.00708800
N	0.60187600	-2.28030300	0.00693900
H	1.11421900	2.70587400	0.00773200

N	2.38618900	-0.67495600	-0.00333800
O	3.27193900	-1.64099700	-0.00054300
O	2.77365200	0.58047800	-0.01041800
N	-2.44585700	1.05003300	-0.00548300
H	-3.32537800	1.55324800	-0.00751300
O	-3.59369100	-0.95230900	0.00209800
O	1.39039500	3.65756900	0.01459000
H	0.52813000	4.10008100	0.02226800
